# Accounting for spatial autocorrelation improves the estimation of climate, physical environment and vegetation’s effects on boreal forest’s burn rates

**DOI:** 10.1007/s10980-017-0578-8

**Published:** 2017-10-24

**Authors:** Jeanne Portier, Sylvie Gauthier, André Robitaille, Yves Bergeron

**Affiliations:** 1 Département des Sciences Biologiques, Centre for Forest Research (CFR), Université du Québec à Montréal, Case postale 8888, Succursale Centre-ville, Montreal, QC H3C 3P8 Canada; 2 Natural Resources Canada, Canadian Forest Service, Laurentian Forestry Centre, P.O. Box 10380, 1055 du PEPS, Stn. Sainte-Foy, Quebec, QC G1V 4C7 Canada; 3 Ministère des Forêts, de la Faune et des Parcs, Direction de la recherche forestière, 2700, rue Einstein, Quebec, QC G1P 3W8 Canada; 4 Forest Research Institute, Université du Québec en Abitibi-Témiscamingue and Université du Québec à Montréal, 445, boul. de l’Université, Rouyn-Noranda, QC J9X 5E4 Canada

**Keywords:** Coniferous boreal forest, Quebec, Eastern Canada, Wildfires, Burn rate, Spatial autocorrelation, RAC models, Autocovariate

## Abstract

**Context:**

Wildfires play a crucial role in maintaining ecological and societal functions of North American boreal forests. Because of their contagious way of spreading, using statistical methods dealing with spatial autocorrelation has become a major challenge in fire studies analyzing how environmental factors affect their spatial variability.

**Objectives:**

We aimed to demonstrate the performance of a spatially explicit method accounting for spatial autocorrelation in burn rates modelling, and to use this method to determine the relative contribution of climate, physical environment and vegetation to the spatial variability of burn rates between 1972 and 2015.

**Methods:**

Using a 482,000 km^2^ territory located in the coniferous boreal forest of eastern Canada, we built and compared burn rates models with and without accounting for spatial autocorrelation. The relative contribution of climate, physical environment and vegetation to the burn rates variability was identified with variance partitioning.

**Results:**

Accounting for spatial autocorrelation improved the models’ performance by a factor of 1.5. Our method allowed the unadulterated extraction of the contribution of climate, physical environment and vegetation to the spatial variability of burn rates. This contribution was similar for the three groups of factors. The spatial autocorrelation extent was linked to the fire size distribution.

**Conclusions:**

Accounting for spatial autocorrelation can highly improve models and avoids biased results and misinterpretation. Considering climate, physical environment and vegetation altogether is essential, especially when attempting to predict future area burned. In addition to the direct effect of climate, changes in vegetation could have important impacts on future burn rates.

**Electronic supplementary material:**

The online version of this article (doi:10.1007/s10980-017-0578-8) contains supplementary material, which is available to authorized users.

## Introduction

Wildfires have been shaping boreal forests for millennia by creating mosaics of landscapes of different age structure, size, and composition (Stocks et al. 2003; Gauthier et al. 2015a). In the north American coniferous boreal forest, the spatial variability of fire regimes has been demonstrated at scales of millennia (Hu et al. 2006; Senici et al. 2015), centuries (Girardin and Mudelsee 2008) and decades (Kasischke and Turetsky 2006). This spatiotemporal variability is decisive for many ecological attributes such as biodiversity (Gauthier et al. 2015a), and societal attributes such as forest management (Johnson et al. 1998). For these reasons, better understanding wildfires constitutes a burning challenge in landscape ecology, especially as their semi-random nature makes them a complex process to study.

A notable issue is the spatial autocorrelation related to the contagious nature of fire spreading which requires appropriate spatially explicit methods (Reed et al. 1998). Indeed, two locations close to each other are unlikely to be independent, which breaks the assumptions of most standard statistical analyses (Dormann et al. 2007). Spatial autocorrelation is often disregarded by fire studies, but this omission can lead to type I error and consequently to incorrect estimation of parameters and important misinterpretation (Reed et al. 1998; Dormann et al. 2007; Mishra et al. 2016).

Fire regimes often vary depending on various environmental factors (Larsen 1997; Hu et al. 2006). Many fire studies in boreal ecosystems attempt to better understand the spatial heterogeneity of fire regimes by investigating top-down effects, such as climate at regional to global scales (Drever et al. 2008; Girardin and Wotton 2009), or bottom-up effects, such as vegetation (Cumming 2001; Terrier et al. 2013) and physical environment (Rogeau and Armstrong 2017) at local to regional scales. Some studies have evaluated the relationship between the spatial heterogeneity of fire regimes and several of these attributes (e.g. Drever et al. 2008; Marchal et al. 2017; Rogeau and Armstrong 2017). However, some uncertainties remain about the contribution of all these factors relative to each other.

The goal of our study was (i) to implement a spatially explicit method involving residuals autocovariate (RAC) models (Crase et al. 2012) in burn rates analyses, and to test its performance against more standard models not accounting for spatial autocorrelation; and (ii) to use this method to determine the relative contribution of climate, vegetation and physical environment to the spatial variability of burn rates in the coniferous boreal forest of eastern Canada. First, we used ordinal logistic models to test for the effects of climate, vegetation and physical environment on the spatial variability of burn rates. Then, in order to account for spatial autocorrelation, RAC models (Crase et al. 2012) were built based on the ordinal logistic models. The extent of the spatial autocorrelation was linked to the fire size distribution of the study area. The relative importance of each group of factors to the variability of burn rates was calculated and their individual effects were identified.

## Materials and methods

### Study area

**Fig. 1:**
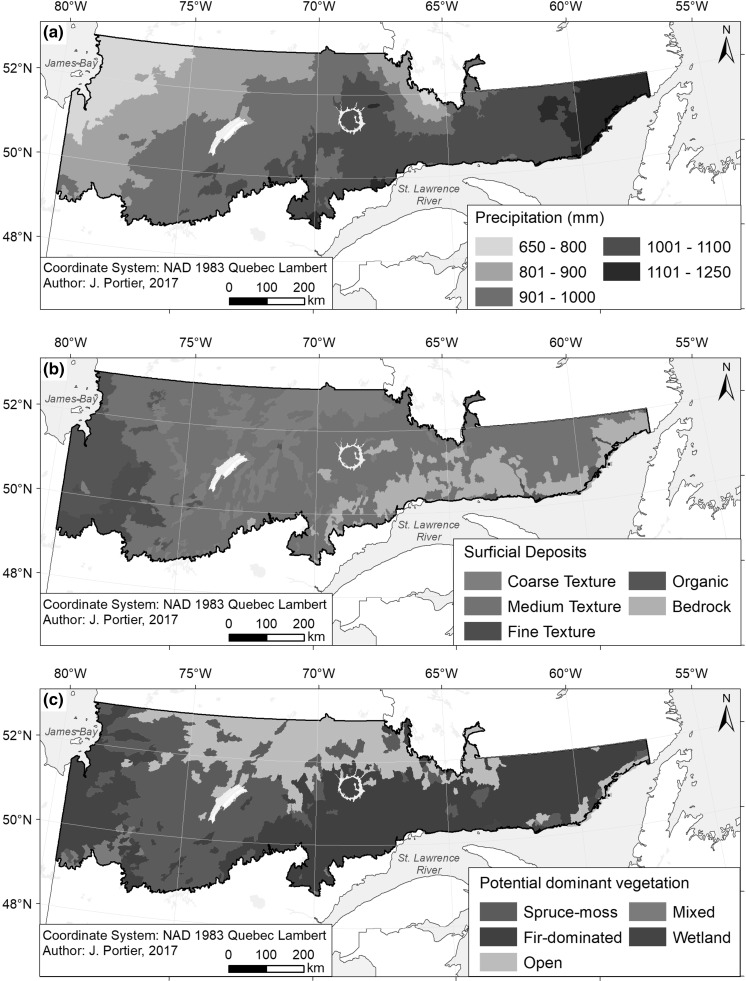
Maps of the study area showing distributions of **a** mean annual precipitation, **b** dominant SD texture, and **c** potential vegetation

The study area is located in the boreal vegetation zone of Quebec, eastern Canada. It covers 482,000 km^2^ and stretches between latitudes 49°N–53°N and between longitudes 79°30′W–57°W. Total mean annual precipitation increases from west to east, and to a lesser extent from north to south, ranging from 651 to 1236 mm (Fig. 1a). The mean annual temperatures vary from − 4.9 °C in the north to 1.6 °C in the south. The topography notably varies across the study area (Robitaille et al. 2015). While the West has a relatively flat topography and low elevation, the north-central portion experiences a higher elevation with a gentle relief. Towards the Southeast, relief is strongly dissected by broad north-south valleys. Further east, highly fractured relief rises gradually from sea level to 1000 m. Magnitudes of relief and elevation then gradually decrease towards the eastern lower north shore region of the Saint Lawrence River. In terms of surficial deposits, thick and thin tills and organic deposits are the most abundant, although an important amount of rock is found in the Southeast (Fig. 1b; Robitaille et al. 2015). Forests are largely dominated by black spruce (*Picea mariana* (Mill.) B.S.P.), but also contain other species in smaller proportions, such as jack pine (*Pinus banksiana* Lamb.), balsam fir (*Abies balsamea* (L.) Mill.), trembling aspen (*Populus tremuloides* Michaux) and white birch (*Betula papyrifera* Marsh.).

Analyses were performed at the scale of Land Districts (LDs) that are “areas of land characterized by a distinctive pattern of relief, geology, geomorphology, and regional vegetation” (Jurdant et al. 1977) and are levels of the Ecological Land Classification Hierarchy developed in Quebec (Robitaille and Saucier 1996). A notable advantage of using LDs is that there is a number of environmental variables available at this level. Our study area contains 1114 LDs, with an average size of 42,700 ha. Three LDs were removed from the dataset because they were almost exclusively composed of large bodies of water (Lake Mistassini, Lake Albanel, and Manicouagan reservoir).

### Data

#### Fire

**Fig. 2:**
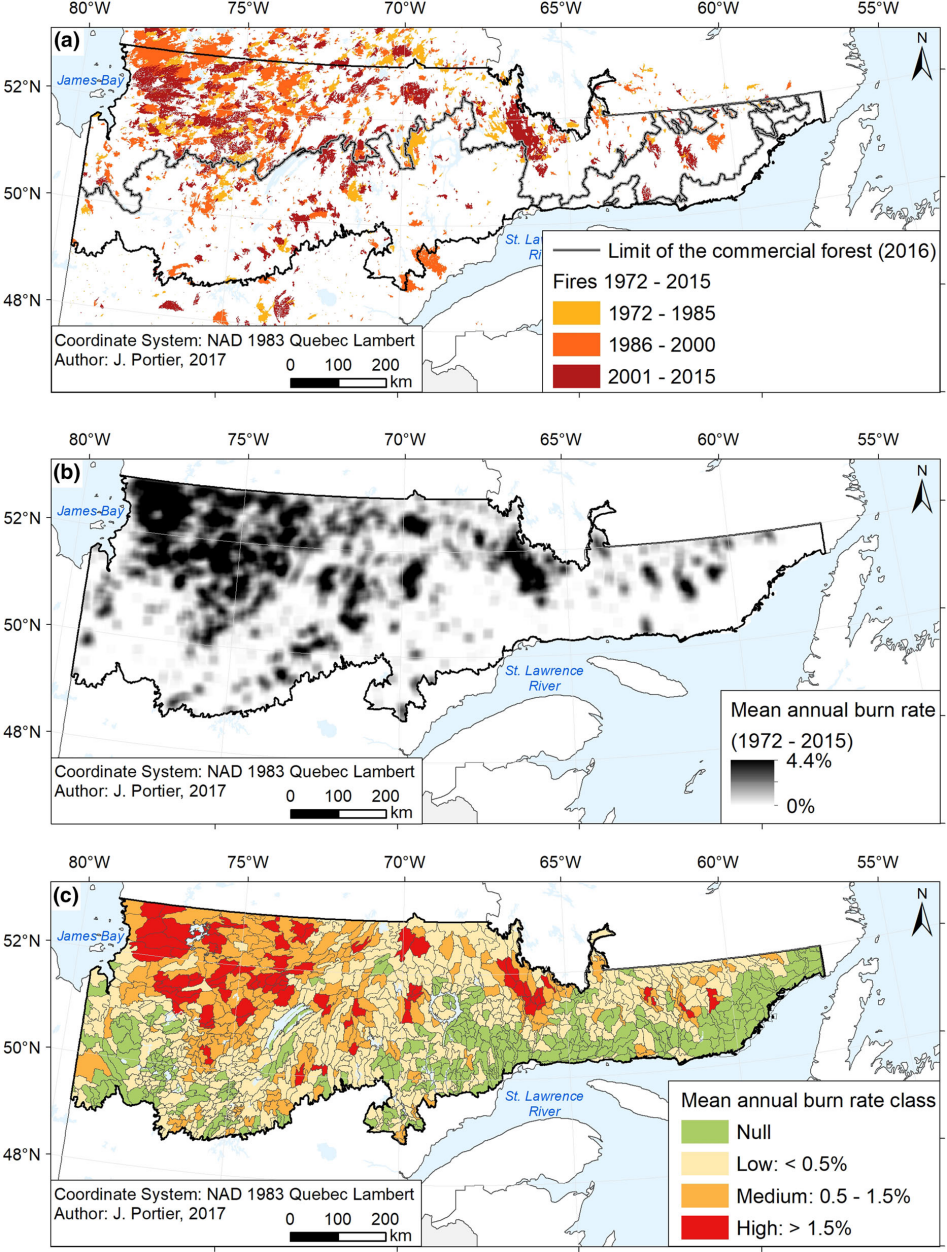
Maps of **a** fires that occurred in the study area during the period 1972–2015; **b** smoothed BRs, and **c** final BR class of LDs

Fire archives obtained from the Ministère de la Forêt, de la Faune et des Parcs du Québec (MFFP) were compiled over the 1972–2015 period (Fig. 2a). All recorded fires were included in the analyses, regardless of their size. South of the limit of the commercial forest established in 2002 (Fig. 2a), data has been submitted to quality control and fire dates are considered more precise (Gauthier et al. 2015b) than in the North where remote sensing techniques have been used to delimitate the boundaries of burns and to determine fire dates. Consequently, a few fires in the North could not be precisely dated, which is why the fire dates have been specified in 5-year intervals (Leboeuf et al. 2012). For those, the middle year of the class was used in the analyses. Minimum, maximum, and mean fire size in the study area were respectively 0.4, 494,340 and 5138 ha. In total, 2079 fires were recorded.

#### Climate

Variables were extracted at each LD’s centroid using the BioSIM 9 software (Régnière and Saint-Amant 2008). BioSIM compensates for the scarcity of weather stations in the study area by interpolating climate data from nearby weather stations, adjusting for elevation, latitude, and longitude (Régnière and Saint-Amant 2008). Climate data was extracted over the 1971–2009 period (Lord 2013). Climate variables included mean annual precipitation (Fig. 1a) and Drought Code (DC) calculated for spring months (May and June) and for the month of July. The DC is part of the Fire Weather Index System and is derived from meteorological observations, namely rainfall and temperature (Amiro et al. 2004).

#### Physical environment

The physical environment was represented by three variables compiled at the LD level: dominant relief, dominant surficial deposit (SD) (Fig. 1b) and percentage of water. Dominant relief and SD refer to the dominant type of relief and SD (i.e., type covering the largest area) in an LD. The dominant relief was classified as either plains and valley bottoms (flat), low hills and hills (minimally rugged) or high hills and mounts (moderately to highly rugged). SDs are an indicator of the drainage potential of the forest floor. This variable was classified based on the texture of the dominant SD, i.e., coarse, medium or fine, except when the dominant SD was organic or when an LD presented mostly bare bedrock at its surface, in which cases the variable was classified as organic or bedrock, respectively. The percentage of water refers to the percentage of an LD covered by lakes and large rivers.

#### Vegetation

Potential vegetation (Fig. 1c) was compiled at the LD level and refers to the dominant type of potential vegetation in an LD. This variable was used as an indicator of the type of fuel theoretically dominating an LD while minimizing the influence of the last disturbances that occurred. Potential vegetation represents a specific tree assemblage that was determined based on physical environment’s characteristics, established vegetation, presence of indicator species, pre-established regeneration, and successional pathways (Grondin et al. 2007). Potential vegetation was grouped into five forest categories: spruce–moss, fir-dominated, open, wetlands and mixed forests. Analyses have also been performed with current vegetation (see Appendix A in Supplementary Material) in order to compare the results with potential vegetation. Contrary to potential vegetation, current vegetation is mainly determined by the recent disturbance history (Grondin et al. 2014). This variable represents the dominant vegetation type that was present in an LD in 2009 (Leboeuf et al. 2012).

### Statistical analyses

#### Burn rates

Compiling burn rates at the LD level was realized using ArcGIS software v10.2.2. First, one grid with a resolution of 1 km × 1 km was built for each year of the 44-year study period. In each grid, each cell was assigned with one if a fire burned part or the entirety of the cell during the year into consideration, or with zero if it did not burn during that year. The grids were then smoothed using a 400 km^2^-window, the approximate mean size of an LD. To achieve this step, each cell was assigned with the mean value of the surrounding 400 cells, corresponding to the proportion of the surrounding landscape that burned during the year into consideration. All 44 yearly grids were then averaged so that each cell showed the mean smoothed annual burn rate (Fig. 2b). The mean annual burn rate (BR) was then extracted at each LD’s centroid and converted to percentages. This smoothing process reduced the bias resulting from the fact that fires do not stop spreading at LDs’ boundaries. Moreover, this method uniformized the area on which BRs were calculated, therefore dealing with potential biases associated with the varying size of LDs.

BRs were then classified into 4 classes representing the recent past natural variability of BRs in eastern Canada (Bergeron et al. 2006): Null (BR = 0; n = 331); Low (BR < 0.5%; n = 486); Medium (0.5% < BR < 1.5%; n = 219); and High (BR > 1.5%; n = 78) (Fig. 2c).

#### Ordinal logistic regression

Statistical analyses were performed using R software v3.3.2 (R Core Team 2016). Ordinal logistic regression was used to test the relationship between BR classes and vegetation, climate and physical environment at the LD level. First, a full model was built containing all variables, on which the proportional odds assumption was verified. Secondly, a backward AIC (Akaike Information Criterion) model selection was realized. In order for a variable to be removed, the AIC value of the model without the variable had to be no greater than two compared with the AIC value of the model with the variable. Once no variable could be further removed, and in case several models were within two delta-AIC of the best model, the most parsimonious model was kept as final model. The AIC of the final model was compared with the AIC of the null model to ensure the overall improvement. Ordinal logistic models were built using the *lrm* function of the “rms” R package (Harrell 2016).

#### Residual autocovariate (RAC) models

Our data cannot be considered independent because of the spatial autocorrelation between LDs. Indeed, two neighboring LDs are more likely to share common characteristics than those further apart, whether it is in terms of area burned because of the contagious way fires are spreading, or in terms of environmental factors. Autoregressive models are widely used to account for spatial autocorrelation in species distribution studies (Lichstein et al. 2002; Dormann et al. 2007), and have shown interesting results in at least one fire study (Mishra et al. 2016). They are built by adding an autocovariate, calculated from the spatial autocorrelation contained in the response variable, as an additional variable to a regular model. It efficiently reduces the bias resulting from spatial autocorrelation that can often lead to biased parameter estimates and increase type I error rates (Dormann et al. 2007; Crase et al. 2012).

Here, we used an extension of the common autoregressive approach, known as the Residuals Autocovariate (RAC) approach (Crase et al. 2012). The autocovariate of a RAC model, derived from the model residuals instead of the response variable itself, represents the strength of the relationship between model residuals at a given location and residuals at neighboring locations (Crase et al. 2012). The advantage of RAC models over usual autoregressive models is that by fitting the autocovariate on model residuals, explanatory variables that are also spatially correlated have a chance to account for the spatial autocorrelation of the response variable. RAC models better estimate the true influence of explanatory variables because the autocovariate only represents the variance resulting from the spatial autocorrelation that is unexplained by these variables (Crase et al. 2012).

**Fig. 3:**
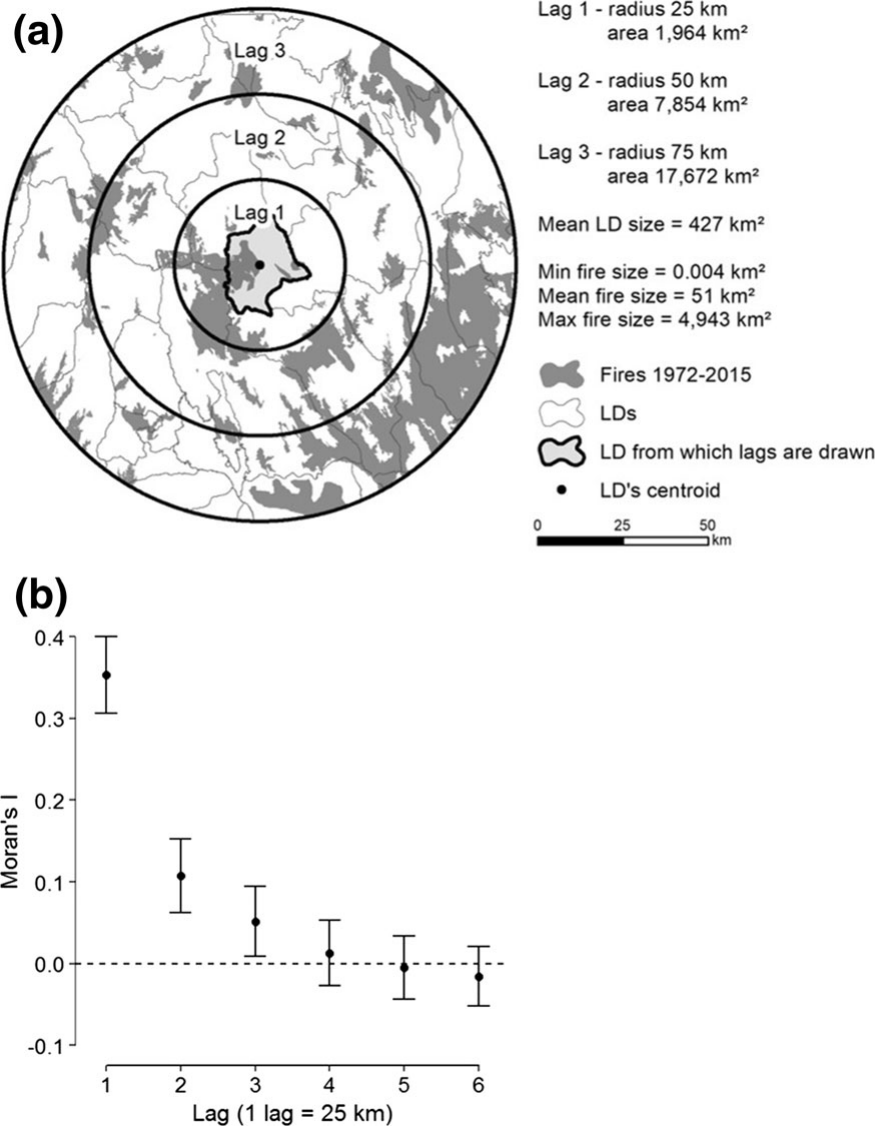
**a** Representation of lags one to three around an LD, as well as fires that occurred over the 1972–2015 period. The LD in this example was chosen because it was the same size as the mean size of LDs. **b** Spatial correlogram calculated on the residuals of the final ordinal logistic model. The correlogram shows Moran’s I associated with each lag as well as their respective Bonferroni-corrected confidence intervals

Several steps were required to build the RAC model. First, a distance matrix was calculated based on the geographic coordinates of LDs’ centroids and the size of a predefined lag using the *dnearneigh* function of the “spdep” R package (Bivand et al. 2016). The lag is defined as the distance between two neighbors when all observations are equally spaced out. As LDs have different shapes and sizes, here we defined lag 1 as the distance at which 95% of the LDs had at least one neighbor, i.e., 25 km (Fig. 3a). Therefore, lag 2 refers to LDs within 50 km, lag 3 to LDs within 75 km, and so on.

Secondly, Li and Shepherd’s residuals were extracted from the final ordinal logistic model. They are well adapted to measuring residuals correlation as they provide a single value per observation and contain directional information (i.e., under- or overestimation) between the observed value and the fitted distribution (Li and Shepherd 2012; Harrell 2016).

Thirdly, a spatial correlogram was built based on the distance matrix and the model residuals using the *sp.correlogram* function of the “spdep” R package (Bivand et al. 2016). The correlogram measures, for different lags, the spatial autocorrelation strength in the residuals with Moran’s I (Legendre and Legendre 1998). Moran’s I is an index ranging from − 1 that indicates strong negative spatial autocorrelation, such as dispersion, to 1 that indicates strong positive spatial autocorrelation, such as clustering. A value of zero means a random pattern with no spatial autocorrelation (Cliff and Ord 1981). In order to test for the significance of the Moran’s I for each lag distance, confidence intervals were computed using a progressive Bonferroni correction (Legendre and Legendre 1998). The Bonferroni-corrected significance level (α’) of the *k*-th lag equals the significance level (α = 0.05) divided by *k*, so that α’ = α/*k* (Legendre and Legendre 1998). This approach was applicable because it requires autocorrelation to be expected in the smallest distance classes.

Fourthly, an autocovariate was calculated for each lag at which the correlogram showed a significant spatial autocorrelation using the *autocov_dist* function of the “spdep” R package (Bivand et al. 2016). One RAC model was built per autocovariate. Finally, a pool of models was compiled, containing the final ordinal logistic model and all RAC models. The model having the lowest AIC value was kept as best model. Spatial autocorrelation in the RAC models’ residuals was assessed to ensure that the inclusion of autocovariates led to residuals independency.

#### Goodness of fit

The goodness of fit of the final RAC model was determined using Nagelkerke’s Pseudo-*R* ^2^. Moreover, its predictive capacity was assessed by calculating the Correct Classification Rate (CCR) (Hosmer and Lemeshow 2000; Nur Aidi and Purwaningsih 2013). The CCR is expressed in percentage and was calculated for the accuracy of the overall model and of each class separately using the following equation (Hosmer and Lemeshow 2000):

1 $$ CCR = \frac{number\,of\,correct\,predictions}{number\,of\,observations} \times 100 $$

#### Variance partitioning

Variance partitioning was used on the best RAC model to determine the relative importance of vegetation, physical environment, and climate in the BR variability. The calculation of exclusive and shared variance of the three groups of factors was derived from the method described by Legendre and Legendre (1998), after being adapted for three groups of factors instead of two. Variance was calculated with McFadden’s *R* ^2^ (McFadden 1974).

## Results

### Model selection

The backward model selection showed that four ordinal logistic models, including the full model, were concurrent candidates to best explain the BR classes of LDs (Table 1). The final model, the most parsimonious, included one variable from the climate group (mean annual precipitation), all variables from the physical environment group (dominant relief, dominant SD and percentage of water), and the vegetation group variable (potential dominant vegetation). Analyses performed with current vegetation instead of potential vegetation produced a similar final ordinal logistic model (Table A1 in Supplementary Material).

**Table 1:** Ordinal logistic models within 2 Δ_AIC_ of best model resulting from the backward model selection process, as well as full and null models

Ordinal logistic models			AIC	Δ_AIC_ with best model
Climate	Physical environment	Vegetation		
Precipitation	**Relief + SD + % water**	**Potential vegetation**	**2255.4**	**0.0**
Precipitation + DC spring	Relief + SD + % water	Potential vegetation	2255.5	0.1
Precipitation + DC July	Relief + SD + % water	Potential vegetation	2255.6	0.2
Full model			2256.8	1.4
Precipitation + DC spring + DC July	Relief + SD + % water	Potential vegetation		
Null model			2735.7	480.3

The model used in the subsequent analyses is in bold type

### Performance of RAC models

The spatial correlogram indicated a significant spatial autocorrelation in the residuals of the final ordinal logistic model at lag one to lag three, i.e., within 25–75 km of the LDs’ centroids (Fig. 3). The correlation was strongest at lag one, weakening as lags increased. The AIC-based comparison between the final ordinal logistic model and the three RAC models (one for each lag at which spatial autocorrelation was significant) showed that the RAC model containing the first order autocovariate (i.e., autocovariate calculated at lag 1) performed best, both in terms of AIC and Nagelkerke’s pseudo-*R* ^2^ (Table 2). RAC models’ Nagelkerke’s pseudo-*R* ^2^ were between 1.4 and 1.5 times higher than that of the final ordinal logistic model (Table 2). The CCR and CCR plus or minus one class of the first order RAC model are presented in Table 3. Analyses performed with current vegetation produced a similar final RAC model as those realized with potential vegetation. However, the AIC value of that model was greater by 19 than that of the first order RAC model factoring in potential vegetation, indicating that the latter performed best (Table A2 in Supplementary Material).

**Table 2:** AIC and Nagelkerke’s pseudo-*R* ^2^ of the final ordinal logistic model and of the RAC models

Models	AIC	Δ_AIC_ with best model	Nagelkerke’s pseudo-*R* ^2^
1st order RAC model	**1862.2**	**0.0**	**0.61**
2nd order RAC model	1918.7	56.5	0.58
3rd order RAC model	1971.7	109.5	0.56
Final ordinal logistic model	2255.4	393.2	0.40

The best model used in the subsequent analyses is in bold type

**Table 3:** CCR and CCR ± one class in percentage showing the accuracy of the overall model and of each BR class separately

	BR class				
	Null	Low	Medium	High	Overall
CCR	67.1	74.9	48.4	25.6	63.9
CCR ± one class	100	99.6	99.1	91.0	99.0

### Effect of climate, physical environment and vegetation on BRs

**Fig. 4:**
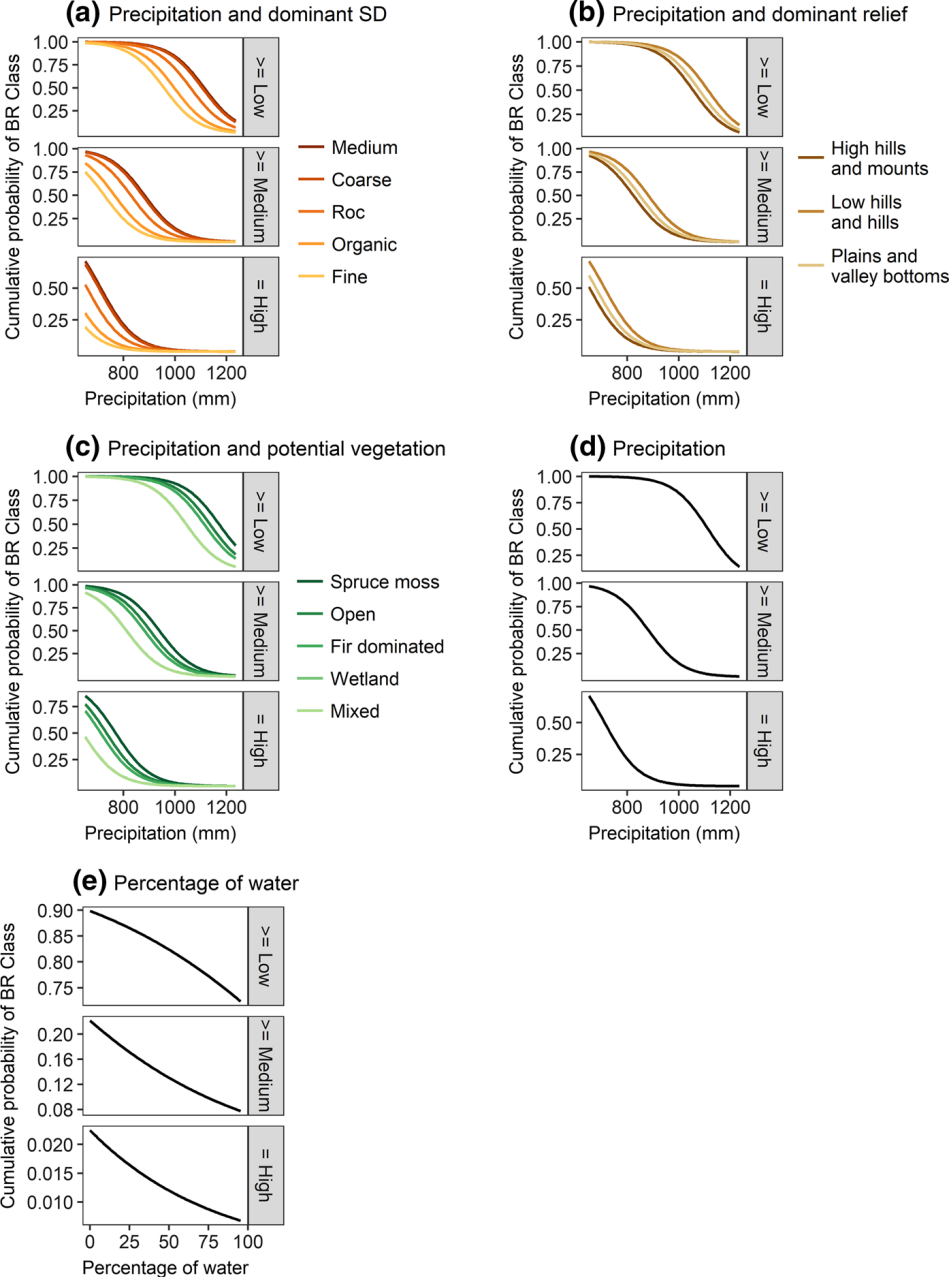
Effects of precipitation and **a** dominant SD, **b** dominant relief, and **c** potential vegetation; as well as effects of **d** precipitation alone and **e** percentage of water alone on the cumulative probability of experiencing at least a low BR, at least a medium BR, or a high BR. In each panel, the continuous variables that are not represented were included in the model’s predictions using their mean value. For dummy variables, the most represented class was used. In panel **c**, the curve representing mixed forests is not visible because it is concealed by the curve representing wetlands

The variables’ effects on BRs were extracted from the first order RAC model. They can be expressed either using odd ratios, i.e., the probability that the BR increases from one class to the next higher one (Table 4), or using the cumulative probability of minimally belonging to a non-null BR class, which is equivalent to the probability for an LD of having at least a low BR, at least a medium BR, or a high BR (Fig. 4). All variables had a significant effect on BRs (Table 4).

**Table 4:** Odd ratios of variables from the first order RAC model and their 95% confidence intervals (95% CI)

	Variables		Odd ratios	95% CI	*p*-values
Climate	Precipitation (for an increase of 1 mm)		0.99	0.98–0.99	< 0.0001
Physical environment	Dominant SD (reference level = Fine texture)	Organic	1.80	0.71–5.00	< 0.0001
		Bedrock	4.61	1.81–12.49	
		Coarse texture	9.07	3.87–22.75	
		Medium texture	10.15	4.61–24.03	
	Dominant relief (reference level = high hills and mounts)	Plains and valley bottoms	1.44	0.88–2.54	< 0.0001
		Low hills and hills	2.37	1.56–3.65	
	Percentage of water (for an increase of 1%)		0.99	0.97–1.00	0.0493
Vegetation	Potential vegetation (reference level = mixed)	Wetlands	1.00	0.26–4.10	< 0.0001
		Fir-dominated	2.83	0.77–13.25	
		Open	3.94	1.18–15.83	
		Spruce–moss	6.67	2.13–27.19	

Odd ratios represent the odds of going from one BR class to the next higher one. Their values are always positive. For instance, for an increase of 1 mm of precipitation, the odds of going from one BR class to the next are multiplied by 0.99, so precipitation decreases the odds of having a higher BR. For dummy variables, the odd ratios are given compared to a reference level. For example, the reference level of the relief variable is high hills and mounts. Therefore, the odds of plains and valley bottoms, and low hills and hills going up one class of BR are respectively 1.44 and 2.37 times greater than those of high hills and mounts. The 95% CI was obtained by bootstrap after 1000 randomizations with replacement of the original dataset and computation of the upper and lower percentiles of the 1000 resulting odd ratios of each variable

First, the probability of belonging to any non-null BR class decreased with increasing precipitation, and precipitation became more limiting as the BR class increased (Fig. 4d). The probability of having a high BR reached a near to zero value when precipitation exceeded 900 mm, while the probability of having at least a low BR was still close to 0.25 in LDs experiencing 1200 mm of precipitation. Secondly, the probability of belonging to any non-null BR class varied with dominant SD (Fig. 4a). LDs dominated by medium and coarse textures had the highest probabilities of belonging to any non-null BR class, followed by those dominated by bedrock, organic, and then fine texture. Thirdly, LDs dominated by low hills and hills had the highest probabilities of belonging to any non-null BR class, followed by those dominated by plains and valley bottoms and then high hills and mounts (Fig. 4b). Fourthly, LDs covered with a high percentage of water tended to have a lower probability of belonging to any non-null BR class (Fig. 4e). Lastly, in terms of vegetation, LDs dominated by spruce–moss forests had the highest probabilities of belonging to any non-null BR class, followed by those dominated by open forests, fir-dominated forests and then wetlands and mixed forests (Fig. 4c). When factoring in current vegetation, LDs dominated by open forests had the highest probabilities of belonging to any non-null BR class. Next were those dominated by wetlands, mixed forests and coniferous-moss forests, all of which showing similar effects on BRs (Fig. A4; Table A4 in Supplementary Material).

### Variance partitioning

**Fig. 5:**
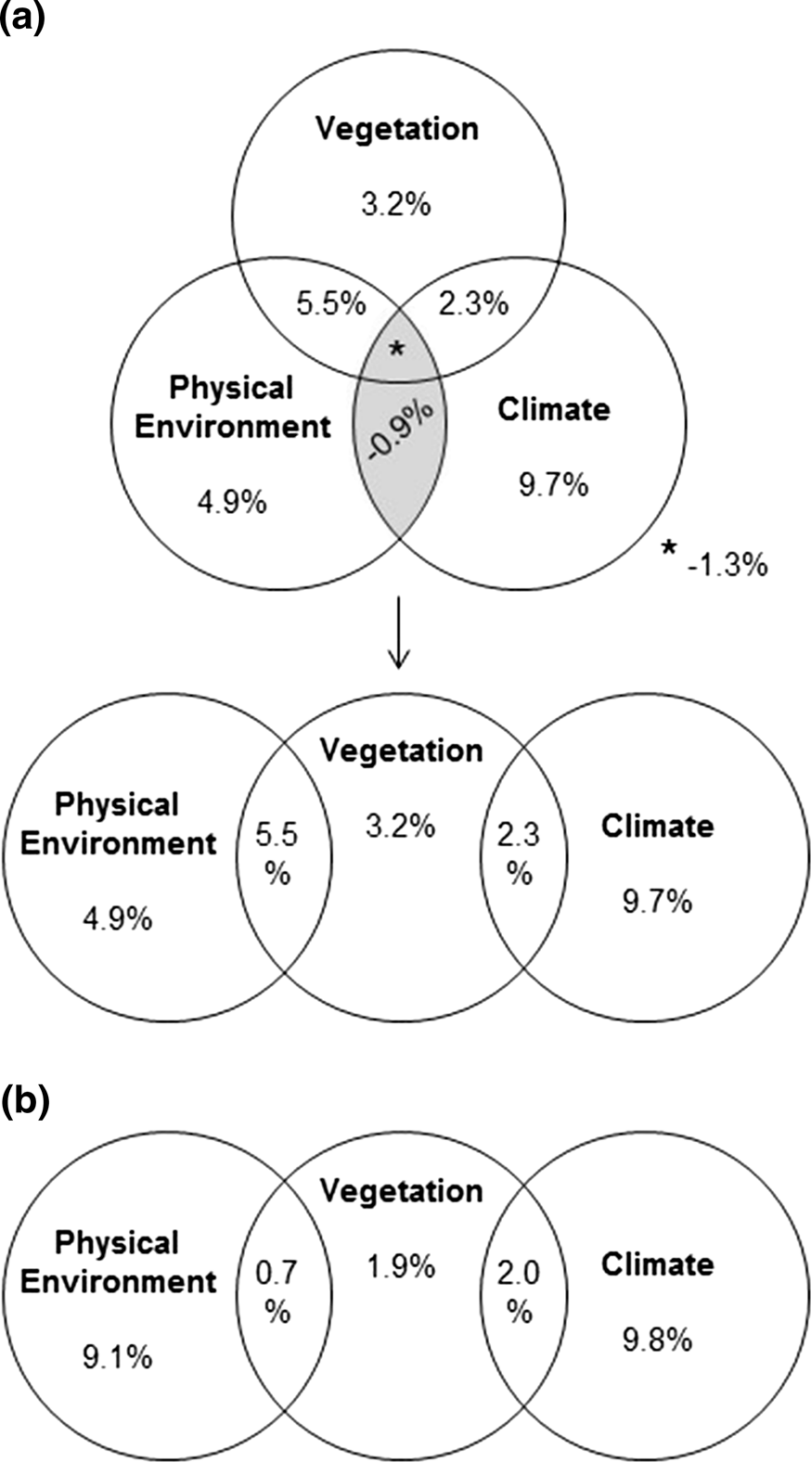
Venn diagrams of variance partitioning of the first order RAC models **a** factoring in potential vegetation and **b** factoring in current vegetation. Variance is calculated as McFadden’s R^2^. The total percentage of variance explained by a given group of factors equals the sum of all percentages within the corresponding circle

Variance partitioning showed that climate, physical environment, and vegetation were responsible for 12.0, 10.4, and 11.0% of variance, respectively (Fig. 5a). Both the vegetation and climate groups, as well as the vegetation and physical environment groups shared a fraction of variance. In contrast, the climate and physical environment groups did not—their shared fraction was negative and close to zero (− 0.9%). This was also the case for the three groups altogether (− 1.3%). A null value indicates that the groups of factors contain no redundant information on BRs, whereas a negative value indicates that the groups of factors together explain the BR better than the sum of the individual effects of these groups (Legendre and Legendre 1998). Therefore, the variance partitioning could be represented by a linear Venn diagram (Fig. 5a). The Venn diagram of the RAC model using current vegetation was similar to that of the model using potential vegetation (Fig. 5). However, the fractions of variance of vegetation alone and shared between vegetation and physical environment were smaller in the case of current vegetation than potential vegetation.

## Discussion

### Importance of taking spatial autocorrelation into account in fire studies

Although rarely accounted for, spatial autocorrelation represents a great issue in fire studies, mainly because fires have a contagious way of spreading (Reed et al. 1998). Consequently, regardless of the scale used in one’s study, fires can spread over two or more units and connect them to each other. We used a smoothing process in the calculation of BRs at the LD level, as well as RAC models as a spatially explicit method in order to control for spatial autocorrelation. RAC models have demonstrated their excellent performance in other fields, such as species distribution modeling (Crase et al. 2012). Although more classic autocovariate models have been used in fire studies (e.g. Mishra et al. 2016), we here report the first use of this RAC method in such study. The RAC ordinal logistic models were found to be a great improvement compared to the corresponding simpler ordinal logistic model, thus underlining the need for taking spatial autocorrelation into account in fire studies (Reed et al. 1998; Mishra et al. 2016). Indeed, our method led to a pseudo-*R* ^2^ 1.5 greater than that of the model that did not account for spatial autocorrelation.

Another advantage of using residuals autocovariates is that it also accounts for the spatial autocorrelation that remains in the explanatory variables after a model was built (Crase et al. 2012). Consequently, the variance partitioning analysis that was based on the first order RAC model was more likely to provide the unadulterated contribution of climate, physical environment and vegetation to the variability of BRs. For instance, we showed that climate and physical environment did not share any fraction of variance, although LDs close to each other were highly likely to share the same climatic and physical characteristics. Without controlling for spatial autocorrelation, a shared contribution—likely related to a type I error—could have been expected, as found by Grondin et al. (2014).

The inclusion of autocovariates calculated at several lags showed that accounting for spatial autocorrelation required to consider LDs that had their centroids up to 25 km apart. The area corresponding to that radius could fit 99.8% of all fire sizes, suggesting that the distribution of fire size is a good indicator of the extent to which data might be spatially correlated. This has great implications for future fire studies, where spatial scales could be partly determined based on the size of fires. For instance, using units larger than the maximum fire size of the study area could reduce the spatial autocorrelation between units. Moreover, fire size is expected to increase in the future in response to the facilitation of fire spread by a more intense and longer drought events (de Groot et al. 2013; Flannigan et al. 2016). As a result, spatial autocorrelation could become an even more important issue in the future, and consideration of the future fire size could be necessary in studies interested in future area burned.

### Factors controlling the BR

Climate, physical environment and vegetation were found to equally contribute to the BR variability, supporting similar conclusions reached by a study conducted in a smaller portion of our study area (Cavard et al. 2015). This also reinforces the importance of considering all these factors together when attempting to predict area burned in boreal ecosystems (Cavard et al. 2015; Marchal et al. 2017). Indeed, exclusively focusing on climate and neglecting the effects of both vegetation (in terms of fuel) and physical environment on fire regimes could lead to highly misleading results (Marchal et al. 2017). While, as previously mentioned, climate and physical environment did not share any fraction of variance, vegetation shared some with both of them.

The fraction of variance shared between vegetation and physical environment was smaller in analyses performed with current vegetation than with potential vegetation. This could reflect the fact that physical environment is a greater determinant of potential vegetation than current vegetation, while the latter mainly results from the recent disturbance history (Leboeuf et al. 2012; Grondin et al. 2014). The fraction of variance brought by vegetation alone was greater and the fit of the model was better when using potential vegetation than current vegetation. This indicates that potential vegetation is a better predictor of the BR variability than current vegetation, partly because it better represents the vegetation that was present before the last fire events.

#### Climate

The importance of weather in driving fires has been demonstrated (e.g. Drever et al. 2008; Cavard et al. 2015), but its role is observed over shorter time periods and smaller spatial scales than those at which our study was conducted. Therefore, the effects of climate on BRs are discussed in this paper in terms of general climatic averages experienced in the LDs. Although different drought indices based on temperatures and precipitation were tested, only mean annual precipitation was retained in the analyses as a climatic variable influencing the BR. This suggests that climatically speaking, the spatial variability of BRs over the 1972–2015 period was mainly driven by precipitation. When falling during the fire season, precipitation leads to moister forest floors and fuel that are less prone to fire spread (Flannigan et al. 2016). On the other hand, high winter precipitation impacts fire regimes by remaining on site for a longer time in spring, taking longer to melt and therefore shortening fire seasons (Westerling et al. 2006).

This result has great implications in a climate change context. The north American boreal zone is expected to experience higher temperatures, changes in the distribution of precipitation throughout the year and increasing annual precipitation in the future (IPCC 2014). However, the increase in precipitation might not be able to compensate for the increasing fuel’s evapotranspiration resulting from higher temperatures (Girardin and Mudelsee 2008; Bergeron et al. 2010; Flannigan et al. 2016). The limiting effect of precipitation being reduced, drier fuels could facilitate fire spread and lead to an important increase in BRs (Amiro et al. 2004; Flannigan et al. 2016). The fire regime could therefore gradually shift towards being controlled by temperatures instead of precipitation. This phenomenon may already be happening in the northwestern part of the study area where the fire regime has intensified since the 1980s (Erni et al. 2016).

#### Physical environment

Physical environment was shown to influence BRs through dominant SD, dominant relief and percentage of water. Previous studies at local scales in eastern Canada have shown that SDs influence fire cycles (Mansuy et al. 2010; Bélisle et al. 2016). At our larger scale, LDs dominated by SDs presenting a coarse or medium texture were the most likely to have a non-null BR, followed by LDs dominated by bedrock, and finally LDs dominated by fine texture SDs or organic deposits. Coarse and medium textures have a high drying potential which leads to dry forest floors that ease fire spread (Flannigan et al. 2016). Although bedrock also has a high drying potential, it usually presents a low vegetation cover due to the absence of soil (Robitaille et al. 2015), and such a limited fuel continuity can reduce fire spread (Murray et al. 1998). Fine texture SDs and organic deposits have an excellent water retention potential and produce moderately to highly wet soils able to slow down or even stop fire spread.

Dominant relief was also shown to affect BRs, with LDs dominated by low hills and hills having the highest probabilities of belonging to any non-null BR class, followed by LDs dominated by plains and valley bottoms and finally by high hills and mounts. Low hills and hills are mostly found on thick till deposits with coarse or medium textures (Robitaille et al. 2015) that facilitate fire spread. In contrast, high hills and mounts are generally found on thin tills and bedrock in rugged landscapes that can act as firebreaks (Bélisle et al. 2016). Moreover, high hills and mounts most often have a higher elevation than the other two relief classes. High elevation areas tend to be subject to lower fire frequency (Rogeau and Armstrong 2017) as they experience shorter fire seasons resulting from lower temperatures and delayed snow melting (Westerling et al. 2006). In addition, there can be a cooling effect from orographic lifting of air masses, leading to increasing relative humidity and eventually precipitation (Rogeau and Armstrong 2017). Lastly, if a few plains and valleys are found in mid- to high elevation, most are located in the low elevation James Bay area. These landscapes are covered with extensive bogs and dominated by fine texture and organic SD (Robitaille et al. 2015), thus preventing fire spread.

#### Vegetation

Vegetation was shown to impact BRs, as suggested by previous studies (Cavard et al. 2015; Boulanger et al. 2017). LDs dominated by spruce–moss forests had the highest probability of belonging to any non-null BR class, followed by LDs dominated by open forests, fir-dominated forests, and then by wetlands and mixed forests. As this probability was lower for LDs dominated by open forests than for those dominated by the denser spruce–moss forests, this suggests that fires need a continuous forest cover for spreading (Murray et al. 1998; Senici et al. 2015). This also confirms previous findings suggesting that boreal forests present a resistance to high BRs, as when stands are open, fires cannot spread because of the lack of fuel, thus inducing a negative feedback between forest cover continuity and fire spread (Héon et al. 2014). Wetlands have an important water retention potential, and often reduce or stop fire spread (Senici et al. 2015; Erni et al. 2016). In the same way, deciduous species that are present in the mixed forests category have been shown to significantly reduce fire risk (Cumming 2001; Terrier et al. 2013).

One distinguishing feature of this study was the use of potential vegetation instead of current vegetation. In fact, we showed that using current vegetation could bias the interpretation of results, mainly because it is highly determined by the recent disturbance history (Grondin et al. 2014). First, recently burned LDs were classified as open in the current vegetation classification. As a result, open forests were suggested to lead to the highest probabilities of belonging to any non-null BR class, which is a misinterpretation of the current vegetation being a cause instead of a consequence of the BRs. This also contradicted the results obtained with potential vegetation which suggested that potential open forests could limit BRs because of their lack of fuel (Héon et al. 2014). Similarly, fir-dominated and spruce–moss forests are combined into a single coniferous-moss forest type in the current vegetation classification, a consequence of the impossibility of distinguishing spruce and fir from photointerpretation. This combined coniferous-moss forest type resulted in the lowest probabilities of belonging to any non-null BR class. However, fir-dominated and spruce–moss forests have been previously shown to be associated with very different fire regimes (Bouchard et al. 2008), corroborating our results from the analyses factoring in potential vegetation. These results reinforce the benefits of using potential vegetation over current vegetation to produce more reliable results concerning vegetation effects on BRs. Although considering the vegetation that burned (i.e. that was present prior to fires) would have been the best way to evaluate the effect of vegetation on BRs, such dataset does not exist. Potential vegetation seems to be the most adequate substitute despite the fact it only is a proxy and therefore could come with some biases.

## Conclusion

We showed that RAC models are an efficient method to account for spatial autocorrelation in fire studies, and that fire size distribution can be used to assess the extent of the autocorrelation. Given the improvements to our models brought by this method, we insist that accounting for spatial autocorrelation in fire studies is highly necessary. Moreover, our results support those of other studies (e.g., Cumming 2001; Cavard et al. 2015; Marchal et al. 2017; Rogeau and Armstrong 2017) that showed that vegetation and physical environment are as important as climate to explain the BR variability in boreal ecosystems. All these factors should therefore be accounted for in fire regime studies, particularly in sight of climate change. For instance, studies attempting to predict future BRs should not only consider future climate, but also possible vegetation changes (Boulanger et al. 2017). Current policies regarding forest management in Canada encourage planners to take fire regime into account in decision making. Our results further support previous studies suggesting that forest management can be used to reduce fire risk (Terrier et al. 2013). Reforestation activities could favor, for example, vegetation less likely to increase BRs in an area already at high burning risk due to its physical environment and climate.

## Electronic supplementary material

Below is the link to the electronic supplementary material.

Supplementary material 1 (DOCX 834 kb)
