# Supplementary material for: Accounting for spatial autocorrelation improves the estimation of climate, physical environment and vegetation’s effects on boreal forest’s burn rates
Source: Landsc Ecol. 2017 Oct 24;33(1):19–34. doi: 10.1007/s10980-017-0578-8 (PMC6954029; doi:10.1007/s10980-017-0578-8)
Supplement: Supplementary file 1 — Supplementary material 1 (DOCX 834 kb) [file 10980_2017_578_MOESM1_ESM.docx]

**SUPPLEMENTARY MATERIAL**

**APPENDIX A. CURRENT VEGETATION**

**Method**

Analyses have also been performed with current vegetation instead of potential vegetation in order to compare the results.

Current vegetation was classified into four forest type – coniferous-moss forests, mixed forests, wetlands and open forests – and represented the dominant vegetation type in an LD in 2009 (Leboeuf et al. 2012). Contrary to potential vegetation, spruce-moss and fir-dominated forests were not distinguished as current vegetation was determined by photointerpretation, which is unable to differentiate spruce from fir. Some LDs were recently burned or cut. In the first case, they were reclassified as open. In the second case, they were reclassified as coniferous moss forest as this is the main type of forests aimed by forest management operations.

**Table A1.** Ordinal logistic models factoring in current vegetation within 2 Δ_AIC_ of best model resulting from the backward model selection process, as well as full and null models. The model used in the subsequent analyses is in bold type.

| **Ordinal logistic models** | | | **AIC** | **Δ_AIC_ with best model** |
| --- | --- | --- | --- | --- |
| **Climate** | **Physical environment** | **Vegetation** |  |  |
| **Precipitation** | **Relief + SD + % water** | **Current vegetation** | **2,252.6** | **0.0** |
| Precipitation + DC July | Relief + SD + % water | Current vegetation | 2,253.2 | 0.6 |
| Precipitation + DC spring | Relief + SD + % water | Current vegetation | 2,253.5 | 0.9 |
| Full model | | | 2,254.8 | 2.2 |
| Precipitation + DC spring + DC July | Relief + SD + % water | Current vegetation |  |  |
| Null model | | | 2,735.7 | 483.1 |

**Table A2.** AIC of the final ordinal logistic model and of the RAC models factoring in current vegetation. The best model used in the subsequent analyses is in bold type.

The CCR of the first order RAC model is 62.9% and its CCR plus or minus one class of CR is 98.3%. Nagelkerke’s pseudo-*R²* of the model is 0.60.

| **Models** | **AIC** | **Δ_AIC_ with best model** | **ΔAIC with 1^st^ order RAC model factoring in potential vegetation** | **Nagelkerke’s pseudo-*R*²** |
| --- | --- | --- | --- | --- |
| **1^st^ order RAC model** | **1,881.2** | **0.0** | **19.0** | **0.60** |
| 2^nd^ order RAC model | 1,885.5 | 4.3 | 23.3 | 0.58 |
| 3^rd^ order RAC model | 1,898.8 | 17.6 | 36.6 | 0.56 |
| Final ordinal logistic model | 2,252.6 | 371.4 | 390.4 | 0.40 |

**Table A3**. CCR and CCR ± one class in percentage showing the accuracy of the overall model factoring in current vegetation and of each BR class separately.

|  | **BR class** | | | | |
| --- | --- | --- | --- | --- | --- |
|  | **Null** | **Low** | **Medium** | **High** | **Overall** |
| CCR | 64.6 | 74.5 | 47.0 | 28.2 | 62.9 |
| CCR ± one class | 99.4 | 99.2 | 99.1 | 85.9 | 98.3 |

**Table A4.** Odd ratios of variables from the first order RAC model factoring in current vegetation and their 95% confidence intervals (95% CI). Odd ratios represent the odds of going from one BR class to the next higher one. Their values are always positive. For instance, for an increase of 1 mm of precipitation, the odds of going from one BR class to the next are multiplied by 0.99, so precipitation decreases the odds of having a higher BR. For dummy variables, the odd ratios are given compared to a reference level. For example, the reference level of the dominant relief variable is high hills and mounts. Therefore, the odds of plains and valley bottoms, and low hills and hills going up one class of BR are respectively 1.39 and 2.56 times greater than those of high hills and mounts. The 95% CI was obtained by bootstrap after 1,000 randomizations with replacement of the original dataset and computation of the upper and lower percentiles of the 1,000 resulting the odd ratios of each variable.

|  | **Variables** | | **Odd ratios** | **95% CI** | ***p*-values** |
| --- | --- | --- | --- | --- | --- |
| **Climate** | Precipitation  *(for an increase of 1 mm)* | | 0.99 | 0.98 – 0.99 | < 0.0001 |
| **Physical environment** | Dominant SD  *(reference level = Fine texture)* | Organic | 0.88 | 0.34 – 2.38 | < 0.0001 |
|  |  | Bedrock | 5.35 | 2.30 – 12.67 |  |
|  |  | Coarse texture | 12.53 | 6.33 – 27.28 |  |
|  |  | Medium texture | 13.86 | 7.74 – 28.75 |  |
|  | Dominant relief  *(reference level = High hills and mounts)* | Plains and valley bottoms | 1.39 | 0.84 – 2.21 | < 0.0001 |
|  |  | Low hills and hills | 2.56 | 1.73 – 3.84 |  |
|  | Percentage of water  *(for an increase of 1%)* | | 0.99 | 0.97 – 1.00 | 0.0468 |
| **Vegetation** | Current vegetation  *(reference level = Coniferous moss)* | Mixed | 1.13 | 0.45 – 2.65 | 0.0001 |
|  |  | Wetlands | 1.25 | 0.37 – 3.93 |  |
|  |  | Open | 2.02 | 1.49 – 2.69 |  |

**Figure captions**

**Figure A1.** Map of current dominant vegetation.

**Figure A2.** Spatial correlogram calculated on the residuals of the final ordinal logistic model factoring in current vegetation. The correlogram shows Moran’s I associated with each lag as well as their respective Bonferroni-corrected confidence intervals.

**Figure A4.** Effects of precipitation and (a) dominant SD, (b) dominant relief, and (c) current vegetation, as well as effects of (d) precipitation alone and (e) percentage of water alone on the cumulative probability of experiencing at least a low BR, at least a medium BR, or a high BR. In each panel, the continuous variables that are not represented were included in the model using their mean value. For dummy variables, the most represented class was used.

**Figure A3.** Venn diagrams of variance partitioning of the first order RAC model factoring in current vegetation. Variance is calculated as McFadden’s R². The total percentage of variance explained by a given group of factors equals the sum of all percentages within the corresponding circle.


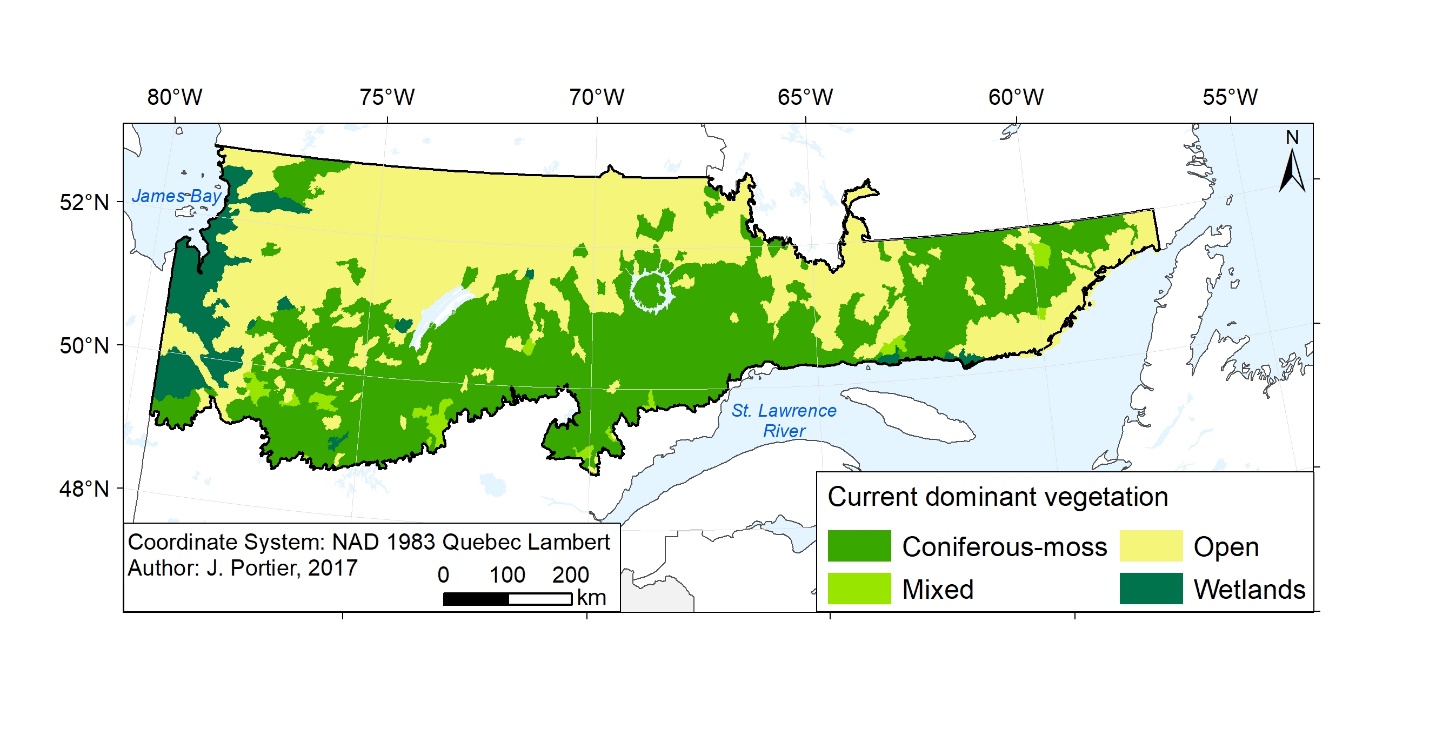


**Figure A1**


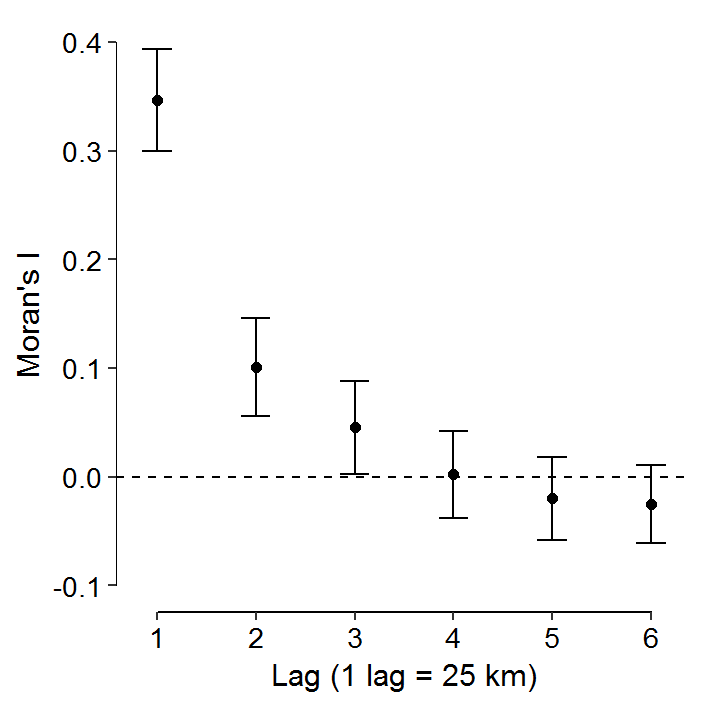


**Figure A2**


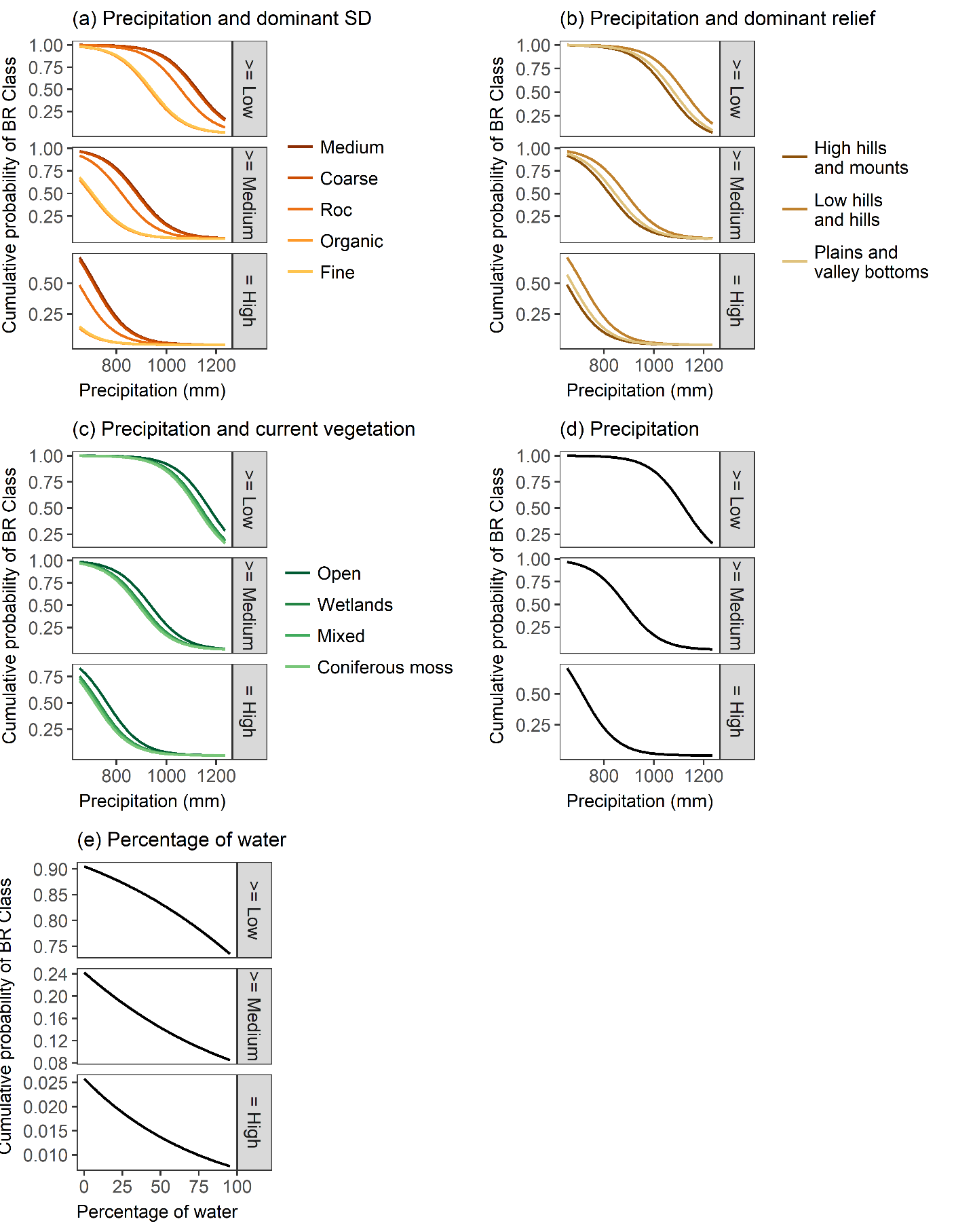


**Figure A3**


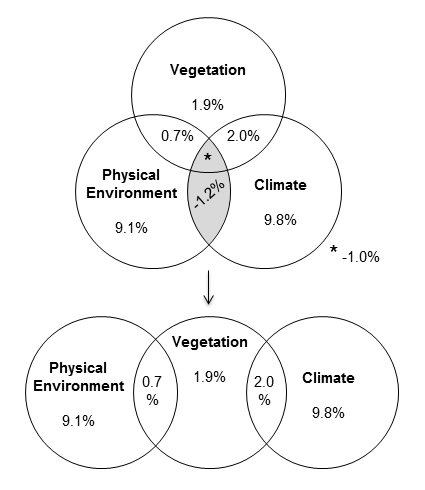


**Figure A4**
